# Assessing the Importance of Content Versus Design for Successful Crowdfunding of Health Education Games: Online Survey Study

**DOI:** 10.2196/39587

**Published:** 2024-02-27

**Authors:** Hong Huang, Han Yu, Wanwan Li

**Affiliations:** 1 School of Information, University of South Florida, Tampa, FL, United States; 2 Department of Applied Statistics and Research Methods, University of Northern Colorado, Greeley, CO, United States; 3 Tandy School of Computer Science, University of Tulsa, Tulsa, OK, United States

**Keywords:** game-based learning, rubrics, Kickstarter, learning game campaign, collaboration, user perception, design, health, learning, gaming, game, evaluation, organization, user, engagement, skill, feedback, assessment, analysis, correlation, crowdfunding, support

## Abstract

**Background:**

Health education games make health-related tasks enjoyable and interactive, thereby encouraging user participation. Entrepreneurs and health educators can leverage online crowdfunding platforms, such as Kickstarter, to transform their innovative ideas into funded projects.

**Objective:**

This research focuses on health education game initiatives on Kickstarter. Through an online user survey, it aims to understand user perceptions and evaluate the significance of 8 distinct components that may influence the success of such crowdfunding initiatives.

**Methods:**

A total of 75 participants evaluated games using 8 dimensions: game rules, learning objectives, narrative, content organization, motivation, interactivity, skill building, and assessment and feedback. The survey data were analyzed using descriptive statistical analysis, exploratory factor analysis, the Wilcoxon-Mann-Whitney test, and multivariate analysis.

**Results:**

Exploratory data analysis showed that, among the 8 dimensions, skill building, content organization, and interactivity were the top-ranking dimensions most closely associated with crowdfunding health education game. The 8 dimensions can be grouped into 3 categories from the exploratory factor analysis: game content–, instruction-, and game design–related components. Further statistical analysis confirmed the correlation between these dimensions with the successful crowdfunding of health education games.

**Conclusions:**

This empirical analysis identified critical factors for game proposal design that can increase the likelihood of securing crowdfunding support.

## Introduction

### Background

Digital strategies, particularly gamification, have introduced a refreshing dynamic to health education [1,2]. Platforms, such as Kickstarter [3], champion these tech-infused health games, providing a unique avenue for their development. By leveraging the power of crowdfunding, Kickstarter and similar platforms facilitate the evolution of health education games. This allows entrepreneurs, educators, game developers, and supporters to access essential resources and connect with audiences eager for meaningful health support and intervention.

### Gamification in Health

Gamification in health integrates game-design elements into nongame health scenarios, aiming to boost user engagement and immersion in health solutions. This transforms routine health tasks into enjoyable, competitive activities. This approach leads to positive behavioral changes, improving overall health, fitness, and adherence to medical treatments and programs [1,2,4,5]. Gamification has been applied to a wide range of medical fields, including health education, medical therapy, obesity, and mental health [1,2,4,5].

Health education games are interactive digital tools specifically designed to impart knowledge or skills related to health and wellness [1,2]. These games transform traditional health-related lessons into enjoyable and engaging tasks, aiming to enhance retention and application of health information in daily life [1,2,4]. Serious health games, created primarily for specific health objectives rather than solely for entertainment, use gaming components to create an educational environment [1,2]. They use gaming components to facilitate a teaching environment, enabling users to learn specific health skills or gain valuable health-related information [1,2]. Especially beneficial for long-term health and chronic-related applications, these games foster positive emotional or empathetic connections among users, leading to improved medical treatment plans and behavior changes [1,2,4].

### The Role of Crowdfunding in Promoting Health Education Games

Given the modest initial investment required and the scale of crowdfunding, it is advocated that crowdfunding serves as a primary method to promote and support the development of health education games. With the recent success of platforms such as Kickstarter, researchers and health care advocates are turning to these tools to fund their projects [6,7]. Through crowdfunding, health educators, entrepreneurs, and other stakeholders can conduct their work to meet community needs while also achieving financial and community outreach goals. This method attracts a varied group of participants who contribute financially, participate in the development, and offer social support [8-12].

Health education games, similar to other game-based learning tools, motivate users by making health-related tasks more enjoyable [1,13]. Online crowdfunding can assist entrepreneurs and health educators with limited resources to translate their innovative ideas into solid and appealing content and formats [14,15]. Crowdfunding platforms help individuals transform ideas into fundable and actionable projects [16,17].

Crowdfunding for health education games benefits users’ self-efficacy, well-being, chronic disease management, and physical activity [9-11]. Rewards, feedback, and socialization elements are frequently used to gamify eHealth in crowdfunding-based health education games. Furthermore, health education games can positively change their health behaviors, benefiting their overall health and wellness [2]. Successful health education crowdfunding projects elicit both intrinsic (altruistic) and extrinsic (rewards and feedback) motivation in order to attract a diverse range of crowdfunding donors, and they work by effortlessly facilitating online digital health engagement [18]. This study aims to explore 8 critical evaluation dimensions from the user’s perspective that influence the success of crowdfunding campaigns for health education games. The findings will guide practitioners and entrepreneurs in strategizing and designing impactful crowdfunding campaigns for health education games.

### Related Works

To understand the intricacies of successful crowdfunding for health education games, we performed a literature review to acquire insights on the various dimensions related to the subject. The literature review enabled us to systematically explore the dynamics of crowdfunding, the principles of game-based learning, and the factors that influence the success of health education games.

#### Dynamics and Success Factors of Crowdfunding Initiatives

To develop and promote content for successful crowdfunding campaigns, extensive planning, outreach, and marketing are required. Data suggest that the most popular crowdfunding projects are those that are creative, participatory, or consumable, such as games, technology, film and video, and art and design [19]. In general, crowdfunding projects have small funding sizes and offer various donor incentives, small gifts, or awards, which leads to a higher success rate for the projects [19]. Such success not only mirrors financial objectives but also nurtures the emergence of communities with shared interests [20]. Numerous game developers have used crowdfunding to fund the initial investment in educational applications [21]. This then encourages more entrepreneurs to participate in collaborative crowdfunding platforms and launch their projects.

Unlike a traditional purchase, crowdfunding involves a high level of social capital influence, particularly the status and reach on social network sites [17]. Social capital creates an online environment that combines collective knowledge, appeal, and emotional responses, enabling investors to make well-informed decisions [17]. This investment process shapes perception and investment behavior. The interaction mechanism has a broader and more pervasive contextual impact, and the crowdfunding campaign design and features also influence decisions [21].

Crowdfunding initiatives require both content richness and ownership diversity [22]. Several studies have explored strategies to optimize the success of such crowdfunding efforts [22,23]. Notably, during crowdfunding, potential investors often evaluate founders based on their personal communication skills and presentation, both of which influence investment decisions [24]. In addition, the use of specific language, the length of campaign text, the frequency of updates, and the inclusion of video in campaign texts have all been correlated with the success of crowdfunding campaigns [25,26]. Reducing the cognitive effort needed to understand campaign content has been shown to result in increased funding [15].

Researchers have also linked crowdfunding success to the trustworthiness and reputation of developers, as well as their experiences on social crowdfunding networks [27,28]. However, the quality of the presented information also plays an important role in determining crowdfunding success [9,29,30]. Factors that contribute to successful crowdfunding factors include the content of the campaign, audience participation, and the timing of fundraising development [31].

#### Health Education Game Development and User Experience

Gamification has been proven to enhance medication and treatment adherence among patients with chronic disease [4,32]. Health serious games, on the other hand, have been praised for their ability to help people with chronic illnesses improve their behavior [2,33]. These games mirror real-life challenges, allowing players to develop coping strategies [17]. They educate players about their condition and the necessary lifestyle alterations, with compelling storylines that ensure better engagement [15,17]. Game interactivity allows players to make decisions, learn from outcomes, and receive feedback on health implications [2,17].

When evaluating the feasibility of a game proposal, it is important to consider both the organization and narrative of the content, as well as the effectiveness of interactive games as a learning tool [34]. A well-organized and clearly written proposal can help the investor understand the purpose, goals, and potential value of the project [31]. Interactive health games can educate users with content and skills [34]. Users can also actively engage with the material, explore and experiment with different concepts and strategies, and receive immediate feedback on their progress [35]. This can help them understand and retain the content and skills being taught.

Game rules and interactivity stand as important components in health game design. Game rules ensure alignment with educational objectives, and the inherent challenge-reward system in these games drives players to continue, thereby continuously learning and adopting healthier behaviors [5,13]. Defining game rules or challenges and delivering feedback can increase users’ self-concept, efficacy, knowledge skills, communication, and social support, resulting in better health behaviors for self-care and adherence, lowering health costs, and establishing a stronger health system [18].

Health education game users are drawn to characters that resemble them, experiencing validation when such characters are featured in media [36]. Young role models, especially those in media genres such as cartoons and video games, are particularly valued by these users [37]. For example, the motivation and design of the interactive health game series can focus on using positive role models to inspire and motivate players [37,38]. These role models are described as being successful in their adventures while also managing their health, which could help users, including children with chronic illnesses such as asthma or diabetes, feel more positive about their own abilities to manage their health and self-care [37,38].

Interestingly, health game players without specific medical conditions are often less certain about in-game decisions compared to their peers with those conditions [37]. Health education games allow players to try new things, fail, learn, and eventually win. Such games also motivate users to adopt a healthier lifestyle, adhere to medical advice when unwell, navigate life crises, and foster close social connections for support [39].

Regarding assessments and feedback mechanisms, health learners who receive personalized feedback and engage deeply with medical content tend to experience great benefit. This approach is especially effective in reaching younger individuals who might not typically consult other media or seek expert health advice [40]. Interactive health games not only foster communication and social support but also empower users to discuss their health with friends, family, and health care professionals. They also motivate users to actively seek out advice and support [37]. For instance, in a series of interactive health games, players accessed factual details about the causes, treatments, social contexts, and self-care options related to specific health topics [37].

#### Game-Based Learning Principles

One of the game-based learning principles that allows users to benefit from the game is the development of problem-solving skills [41], and educational games can assist users in developing these skills [41,42]. The modalities of game content representation should be adjusted to boost motivation and performance [43]. If learners cannot understand the app’s content, no matter how rich and useful it is or how beautiful the design is, the app’s entire instructional value is lost [44]. Learners can learn problem-solving, strategic and analytical thinking, decision-making, and other 21st century skills in narrative-centered learning environments [45].

Based on the constructivist learning theory, individuals gain deeper insights about the world through direct experiences and interactions [46,47]. Games offer a dynamic and interactive environment that aligns with this theory, enabling learners to actively explore, experiment, and tackle challenges [46,47]. The appeal of a game’s narrative indicates its potential to captivate users [48]. The game creators should focus more on the content, storyline, and interaction components of the game to attract individual users when determining whether it will be successful or not [48].

The quality of a learning game is significantly influenced by the effectiveness of user feedback [49,50]. Numerous studies have shown that feedback enhances learning outcomes [51]. It provides learners with clarity on their strengths and areas that need improvement; it also serves as a motivational tool, encouraging continuous learning even within the gaming context [51].

Educational games can customize learning experiences by gauging a student’s readiness, providing constructive feedback, and modifying the level of challenge [52]. It is essential for an educational game to have well-defined learning objectives that detail the desired skills and knowledge [53]. Game rules facilitate learning by allowing players to interact with their environment [54]. Achieving these objectives depends on adhering to specific rules, which may involve certain challenges or conditions that the learner must satisfy [43].

A learner’s level of motivation can greatly influence their enthusiasm or indifference toward a task [55,56]. Moreover, there is substantial evidence suggesting that motivation enhances cognitive functions, particularly influencing what learners focus on and how they assimilate information [57-59].

Literature suggests that multiple factors influence the success of crowdfunding campaigns, especially those related to health education games [51,54]. These range from the trustworthiness of the developers and the quality of information presented to the design and content of the game itself. Although previous studies have shed light on the general principles of game-based learning and the dynamics of crowdfunding, there remains a gap in understanding how these principles specifically apply to health education games on platforms such as Kickstarter. Moreover, the user’s perspective, which is crucial in determining the success of such campaigns, has not been thoroughly explored. We aim to bridge this knowledge gap by focusing on the user’s perception and evaluating the critical components that resonate most with potential users, thereby influencing the success of health education game initiatives on crowdfunding platforms.

### Objectives

This study aims to provide a comprehensive overview of 17 health education game projects launched on the crowdfunding platform Kickstarter and to understand user perceptions concerning the important factors that determine the success of such health education game crowdfunding initiatives. To achieve this, we conducted a user survey using a health education assessment rubric specifically designed to evaluate the key components contributing to the success of these projects on Kickstarter.

## Methods

### Data Collection for Health Education Games

A comprehensive keyword search using “Health, Education, Learning, Game” was conducted in August 2019 on Kickstarter, which identified 17 online health education game projects (Table 1). On the Kickstarter site, the system marked a project as “Successful” if it met or exceeded its financial goal within the time set by the creators. Conversely, projects that failed to meet their financial target within the designated period were labeled as “Unsuccessful” (Table 1).

**Table 1.** Descriptive data of health education game projects from the crowdfunding site Kickstarter. A project’s success on Kickstarter was determined by its ability to achieve its financial goal within the set time frame.

Health education game	Pledge (US $)	Goal (US $)	Backer count, n	Country	Successful^a^
Playout: The Exercise Card Game [60]	11,011	10,000	224	United States	Yes
ACLS MegaCode Simulator for health care professionals [61]	328	1997	8	Canada	No
Blush by Renaissance [62]	5065	3500	80	Canada	Yes
Body Cycle Health Education App [63]	1778	20,000	41	United States	No
CHiLD - a psychological 2D RPG [64]	1199	554	92	Norway	Yes
Destiny’s Sword for mental health [65]	30,930	30,000	209	Canada	Yes
Facing Dragons: a mixed-reality game to unlock your purpose [66]	3361	7104	34	Canada	No
Freestyle Jam Camp [67]	1145	500	18	United States	Yes
Mobile games to quantify symptoms of mental health disorders [68]	127	450,000	5	United States	No
PRESCRIPTION Playing Cards [69]	30,420	7500	178	Canada	Yes
Talk to Me visual novel: mental health [70]	4977	4460	146	United States	Yes
TEN: a card game designed to promote brain health [71]	1445	14,000	39	United States	No
The Chakra Collectable Coin [72]	1682	1300	41	United States	Yes
The Woosah Kit: a mental health first aid [73]	41	6236	3	United Kingdom	No
Tournesol Kids Game: activity cards to build resilience [74]	10,435	5000	140	United States	Yes
Youth Run The World 5K [75]	7370	7000	74	United States	Yes
Zombied: gamify health and fitness activities [76]	12	37,217	2	United Kingdom	No

a "Yes” refers to “Successful” projects that met or exceeded their financial goal, whereas “No” refers to “Unsuccessful” projects that did not.

### Ethical Considerations

Before commencing this study, the researchers obtained approval from the Institutional Review Board of the University of South Florida (001588). The participants provided informed consent, with the option to withdraw at any time without penalty. The Institutional Review Board approval sufficiently covered the secondary use of data. The study guaranteed that all collected data were either anonymized or deidentified to protect personal information, with stringent protective measures in place for any data that could not be fully anonymized. The study was voluntary, without any compensation for participation.

### Online Survey Design

We use the Qualtrics online survey platform (Qualtrics) to create an online survey based on health education game assessment rubrics derived from the literature. This survey allowed participants to evaluate and rank crowdfunding health education games on the Kickstarter website. The survey incorporated 8 dimensions—each essential for the evaluation of health education games. These dimensions, along with their definitions and cited literature, are presented in Table 2.

**Table 2.** Crowdfunding health education game evaluation dimensions and definitions.

Dimensions	Definition	Related literature
Skill building	The game’s ability to progressively impart and reinforce health-related skills to players, ensuring that learning is continuous and effective throughout the game’s duration.	[42,77]
Content organization	The clarity, structure, and logical flow of the game’s health education content, ensuring that it is presented in a manner that is both comprehensible and engaging for players.	[35,53,78]
Narrative	The clarity and continuity of the game’s storyline in relation to health education, ensuring that players experience a coherent sense of progression and purpose as they navigate through the game’s content.	[25,48]
Interactivity	The game’s ability to facilitate effective interactions, the completion of health-related tasks, and active participation through user-driven inputs and actions.	[35,77,79]
Assessment and feedback	The game’s capability to immediately evaluate and communicate a player’s progression and provide timely and relevant feedback.	[35,80-82]
Game rules	The game provides clear, concise, and easily comprehensible rules to the players.	[35,52,83,84]
Learning objectives	The game delineates specific, measurable outcomes that players are anticipated to achieve upon its completion.	[35,85-87]
Motivation	The game’s elements are intriguing and appealing enough to prompt user participation and action.	[88-90]

Before the main survey was launched, a pilot test of the survey instrument was conducted with 7 undergraduate students majoring in health science. This pilot test aimed to assess the validity and understandability of the survey questions. The participants were asked to read through the survey and provide feedback on its clarity and relevance. Based on their comments, necessary revisions were made to the questions to enhance the overall quality of the survey.

In the final version of the survey, participants rated the dimensions on a 3-point Likert scale. The scoring system for these dimensions ranged from 0 to 2, with the following interpretations: 0=“Does not meet expectations” or “Poor,” 1=“Meets expectations” or “Fair,” and 2=“Exceeds expectations” or “Good.” Participants could also select “Unable to decide” or “Not applicable” if they felt unable to make a judgment on a particular dimension. Additionally, an open-ended question was incorporated: “Do you have any comments or concerns (accuracy of terms, comprehensiveness, clarity of questions, etc) for this question sets?” This allowed participants to provide further feedback on the survey questions.

In November 2019, undergraduate students majoring in health science were invited to participate in the online survey. Those who agreed to participate were provided with a standardized set of questions, accompanied by comprehensive instructions and definitions for the 8 evaluation dimensions, as detailed in Table 2. Each student was then randomly assigned 1 specific crowdfunding health education game from a pool of 17 games, referenced in Table 1. Their task was to evaluate their assigned game based on these 8 dimensions. Ultimately, 75 undergraduate students were recruited as participants.

### Data Analysis

We used STATA 15 software (StataCorp) for statistical analyses. We used several data analysis approaches to understand the results.

#### Descriptive Statistical Analysis

This method provides a summary of the main aspects of the data, offering a simple overview of the data. By calculating the percentage of ranking types and the mean scores of the dimensions, we can gain insights into the general behavior and preferences of the survey participants.

#### Exploratory Factor Analysis

Exploratory factor analysis is used to reduce the data’s dimensionality and identify the underlying relationships between the measured variables [91]. It was used to group the 8 dimensions into meaningful categories, helping to decipher any latent structures within the data set. This ensured that we could identify which sets of dimensions tended to co-occur or were rated similarly by participants.

#### Wilcoxon-Mann-Whitney Test

The Wilcoxon-Mann-Whitney test [92] is a nonparametric statistical hypothesis test used to compare 2 unrelated samples. This test was used to determine if there were any significant differences in the rankings given by participants to different game dimensions.

#### Multivariate Analysis

The aim of this study extends beyond merely understanding the dimensions. It also seeks to predict the success of crowdfunding health education games based on these dimensions. We used logistic regression with a binary variable—success of the crowdfunding project—for prediction [91]. This model can determine the odds of a game being successful based on the rankings of its dimensions, offering insights into which dimensions are the most influential predictors of success.

By using these methods, the study ensured a comprehensive analysis of the data—from understanding the basic patterns and deciphering underlying component structures to finally being able to predict the success of crowdfunding health education games based on their dimensions.

## Results

A list of health education games launched on Kickstarter is presented in Table 1. This table enumerates 17 distinct health education games originating from various countries, namely the United States, Canada, Norway, and the United Kingdom. Some projects have exceeded their goals by a large margin, whereas others have fallen substantially short. The diversity of the sample provides a comprehensive foundation for our study. This diversity enabled an exploration into users’ perceptions regarding educational game assessment rubrics. Such an investigation can discern potential factors that could influence the success trajectory of health education games on crowdfunding platforms such as Kickstarter.

Table 2 focuses on the various dimensions relevant to the design and evaluation of games. These dimensions were based on established literature, highlighting their credibility and validity. When assessing potential predictors of crowdfunding success based on feedback from 75 survey participants, certain dimensions stood out as being more important (Table 3).

**Table 3.** Ranking of the 8 assessed dimensions for crowdfunding health education games (n=75).

Dimensions	Score, mean (SD)^a^
Skill building	1.77 (0.54)
Content organization	1.7 (0.52)
Narrative	1.51 (0.69)
Interactivity	1.51 (0.75)
Assessment and feedback	1.49 (0.69)
Game rules	1.47 (0.71)
Learning objectives	1.39 (0.64)
Motivation	1.29 (0.59)

a Scoring system: 0=”Poor,” 1=”Fair,” and 2=”Good.”

Skill building was ranked first, followed by content organization and then narrative. Skill building holds the top rank due to its emphasis on continuous learning and engagement, ensuring that players progressively acquire and refine their skills throughout the game (Table 3). The importance of content organization is highlighted by its role in enhancing user experience; a well-organized game offers clear navigation, allowing players to immerse themselves fully (Table 3). Narrative further enhances the gaming experience by introducing an engaging storyline that lends context and purpose, enriching the gameplay. Interactivity is important for keeping players engaged. It gives them a sense of belonging and influence within the game world. Yet, intriguingly, motivation ranks the lowest among these dimensions, even though its presence ensures that games are compelling enough to retain players’ interest and drive continuous participation (Table 3). Although skill building and content organization seem to be the areas where these games excel, motivation appears to be a challenging area for many developers.

To identify the assessment structure for campaign initiatives’ quality reflected by 75 survey respondents’ rankings, the study conducted an exploratory factor analysis using principal-components analysis as the extraction method and varimax with Kaiser normalization as the rotation method (Table 4). The cutoff size for criterion loadings was set to 0.45 [59]. Both the Bartlett (*χ*^2^=68.26, *P*<.001) and measure of sampling adequacy (0.57) tests for the sample pointed to a significant level of correlation among the dimensions.

**Table 4.** Factor components for the 8 dimensions in crowdfunding health education games. Principal-components analysis served as the extraction method, and varimax with Kaiser normalization served as the rotation method.

Dimensions	Component		
1	2	3
Game rules	−0.050	−0.057	0.843^a^
Learning objectives	0.253	0.727^a^	−0.025
Narrative	0.181	0.629^a^	−0.335
Motivation	0.665^a^	0.102	−0.021
Interactivity	0.489	0.185	0.506^a^
Skill building	−0.136	0.727^a^	0.266
Assessment and feedback	0.883^a^	−0.036	−0.019
Content organization	0.490^a^	0.370	0.033

a Values above the cutoff size for criterion loadings (0.45).

The exploratory factor analysis indicated that these 8 dimensions can be grouped into 3 components: game content (content organization, motivation, and assessment and feedback), instruction (learning objectives, narrative, and skill building), and game design (game rules and interactivity; Table 4). The game content–related components suggests that a well-organized game with clear feedback mechanisms can effectively motivate players. The instruction-related components reflect the instructional journey of the player, from understanding the objectives and engaging with narrative to building skills. The game design–related components are fundamental to the gameplay experience, ensuring that players are not just passive observers but active participants.

To review the perception gaps among these dimensions for successful or unsuccessful crowdfunding campaigns, group-based comparison was conducted between these dimensional means. Table 5 showed the gaps between successful and unsuccessful games in dimension ratings. Among them, motivation, interactivity, game rules, and learning objectives demonstrated larger difference gaps in decreasing order, and these were followed by assessment and feedback, skill building, narrative, and content organization.

**Table 5.** Wilcoxon-Mann-Whitney test of the 8 assessments based on successful or unsuccessful crowdfunding of health education games.

Dimensions and categories		Answers, n	Score, mean (SD)	*U* statistic	*P* value
**Content organization**				0.28	.78
	Success	53	1.68 (0.55)		
	Unsuccessful	15	1.67 (0.49)		
**Interactivity**				2.05	.04^a^
	Success	53	1.57 (0.72)		
	Unsuccessful	15	1.13 (0.83)		
**Skill building**				0.94	.35
	Success	53	1.79 (0.49)		
	Unsuccessful	15	1.60 (0.74)		
**Learning objectives**				2.03	.04^a^
	Success	51	1.43 (0.64)		
	Unsuccessful	15	1.07 (0.59)		
**Narrative**				.09	.37
	Success	53	1.53 (0.70)		
	Unsuccessful	14	1.36 (0.74)		
**Motivation**				2.91	.004^a^
	Success	53	1.38 (0.56)		
	Unsuccessful	15	0.87 (0.52)		
**Game rules**				2.14	.03^a^
	Success	53	1.55 (0.70)		
	Unsuccessful	15	1.13 (0.74)		
**Assessment and feedback**				1	.32
	Success	53	1.49 (0.64)		
	Unsuccessful	14	1.29 (0.73)		

a Significant level *P*<.05.

The Wilcoxon-Mann-Whitney test comparing distributions of successful and unsuccessful games showed that motivation (*P*=.004), game rules (*P*=.03), learning objectives (*P=*.04), and interactivity (*P*=.04) showed statistically significant difference among these 2 groups (Table 5). These dimensions showed clear distinctions between successful and unsuccessful games, suggesting that these dimensions might be crucial for the success of such games. On the other hand, dimensions such as content organization and skill building, while important, did not show a significant difference between the 2 categories of games. This could mean that both successful and unsuccessful games have well implemented these dimensions, but they might not be the distinguishing factors for success. The multivariate analysis showed that learning objectives and motivation were 2 significant dimensions associated with successful health education game crowdfunding campaigns (Table 6). This suggests that these 2 dimensions might be especially important for the success of health-related games.

**Table 6.** Multivariate logistic regression predicting the success of the health educational games.

Dimensions	Odds ratio (95% CI)	*P* value
Game rules	3.24 (0.68-18.40)	.13
Learning objectives	3.55 (1.42-14.38)	.02^a^
Narrative	1.57 (0.37-6.61)	.54
Content organization	0.07 (0.01-1.55)	.09
Motivation	3.05 (1.46-9.36)	.03^a^
Interactivity	1.70 (0.46-6.22)	.42
Skill building	1.31 (0.21-8.18)	.77
Assessment and feedback	1.38 (0.21-8.88)	.73

a Significant level *P*<.05.

**Figure 1.**
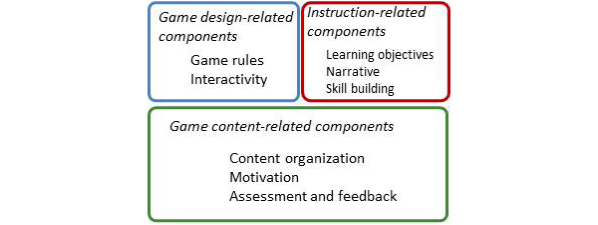
Framework for successful crowdfunding of health education games.

Figure 1 presents an empirical framework that outlines the key components underpinning the success of health education game crowdfunding. The model highlights the balance between foundational structural components, such as game rules and content organization, and experiential elements that enhance the player’s immersion and engagement, such as motivation and narrative. A successful educational game should seamlessly integrate all these facets. This not only ensures the delivery of educational content but also fosters an environment where players are intrinsically driven to remain engaged and continue their learning journey within the game.

## Discussion

### Principal Findings

The crowdfunding landscape for health education games is diverse, with success determined by a myriad of factors beyond just a funding goal. Factors such as the clarity of the project’s purpose, its presentation, and its marketing likely play a substantial role in attracting users [35,41]. It is also important to have a reasonable and attainable goal, as this might increase the likelihood of a project’s success.

Crowdfunding backers, especially on platforms such as Kickstarter, often support projects that offer value beyond just entertainment. Skill building in games implies that players will acquire new abilities or knowledge, making them both fun and beneficial. This dual-purpose might appeal to game players who see an opportunity for a return on investment, not just in potential product rewards but also in personal or societal skill development.

The ranking of these dimensions sheds light on the preferences and priorities of both backers and players. It is possible that backers perceive tangible attributes such as skill building and content organization as immediate indicators of game quality and potential success. These elements can be readily demonstrated in promotional materials, making them more attractive to potential backers. On the other hand, motivation, being more abstract and subjective, might be harder to convey and measure, leading to its lower ranking. It is essential for game developers to recognize these perceptions and strike a balance in their design, ensuring a comprehensive and engaging game experience that appeals to a broad audience.

For skill building, it is essential for players to acquire and build skills as they progress in the game. This ensures continuous learning and engagement. Well-structured game content helps players navigate and understand the game better, thus enhancing their experience. An engaging storyline provides context and purpose, making gameplay more meaningful. Player interactivity is vital for player engagement. Players should feel that they are part of the game world and can influence it. Immediate feedback helps players understand their progression and areas of improvement. Clear rules ensure that players can easily understand how to play the games, leading to smoother game experiences. For health education games, it is important to have clear learning outcomes that guide the game design. The game must be engaging enough to keep players interested and motivated to continue.

The multivariate analysis identified learning objectives and motivation as the 2 significant predictors of a health education game’s crowdfunding success, as detailed in Table 6. This indicates the emphasis users place on clear educational outcomes and the motivation to engage with the game. Users prioritize games that offer clear educational outcomes and that effectively motivate players to engage. The significance of learning objectives suggests that backers might prioritize games that have a clear educational goal, ensuring that players gain tangible knowledge or skills. Motivation, on the other hand, ensures that players remain engaged and committed to the game’s objectives. When combined, these dimensions can lead to a game that not only educates but does so in a compelling manner, maximizing player retention and learning outcomes.

### Limitations and Future Work

The study has some limitations due to the examination of user perception, which is based on a small number of user responses in a small number of crowdfunding campaigns. The study examined subjective opinions across 8 evaluation dimensions, but the reasons for crowdfunding’s effectiveness in health education games require further investigation. In addition, we surveyed participants as potential backers. A more comprehensive approach would involve surveying actual backers, those who make real investments, to discern any differences in perceptions. This could provide a richer understanding of the dynamics at play. The impact of quality on the campaign content and media aspects, as well as user indicators of motivation and interactivity, was investigated in this study. Through crowdfunding, health education games improve engagements, learning components, and cultural adaptability for user engagement [8-10].

### Conclusion

Crowdfunding for health education games presents a unique opportunity to bridge the gap between game developers and potential users. There has been little research that has provided empirical evidence for evaluating user perspectives on crowdfunding health education games. Further empirical evaluations are clearly beneficial to providing a rigorous validation of gamification’s effectiveness in eHealth. This research conducted an exploratory study and identified 3 major components that matter for health game crowdfunding success. These components are related to game design, instruction, and game content. Interestingly, motivation and assessment and feedback were grouped into game content categories, not into game design categories. This indicates that the proposals for health-related crowdfunding education games are comprehensive, encompassing content that is engaging, interesting, and attractive, with solid assessment and feedback components. Among them, given the nature of health subjects, entrepreneurs and educators should pay more attention to game development factors such as motivation, interactivity, and game rules, so that the health or scientific subjects can be easily infused in the gaming process. Making health games look playful and attractive enables users to easily grasp basic health knowledge during the gaming process [93]. Interestingly, there is little difference in content organization between successful and unsuccessful games, which indicates that even if the game content is easy to follow, it is still not enough. Backers and potential funders or users mostly agree with the health content itself, but they care more about the game development components, using these dimensions to assess the crowdfunding game proposal and determine if these game designs are acceptable and make logical sense.

Our findings recognize the importance of aligning game design with user preferences. The success of health education games on crowdfunding platforms relies on a combination of clear educational objectives, effective player engagement mechanism, and well-structured game content. The study highlights the significance of learning objectives and motivation as key determinants of crowdfunding success for health education games. Game developers aiming for success in this domain should prioritize these dimensions, thus ensuring that their games offer a clear educational outcome.
